# ﻿Freshwater fungi in the karst plateau wetlands from Guizhou Province, China: taxonomic novelties in Melanommataceae (Pleosporales)

**DOI:** 10.3897/mycokeys.113.140684

**Published:** 2025-02-07

**Authors:** Ling-Ling Liu, Yong-Xiang Liu, Ya-Ya Chen, Jiu-Lan Gou, Feng Chi, Yi Liu, Xiao-Feng Gu, Quan-Quan Wei, Meng Zhang, Zuo-Yi Liu, Si Zhou

**Affiliations:** 1 Guizhou Provincial Institute of Soil and Fertilizer, Guizhou Academy of Agricultural Sciences, Guiyang, China Guizhou Key Laboratory of Agricultural Biotechnology, Guizhou Academy of Agricultural Sciences Guiyang China; 2 Guizhou Key Laboratory of Agricultural Biotechnology, Guizhou Academy of Agricultural Sciences, Guiyang, China Guizhou Provincial Institute of Soil and Fertilizer, Guizhou Academy of Agricultural Sciences Guiyang China; 3 Guizhou Provincial Institute of Crop Germplasm Resources, Guizhou Academy of Agricultural Sciences, Guiyang, China Guizhou Provincial Institute of Crop Germplasm Resources, Guizhou Academy of Agricultural Sciences Guiyang China; 4 Guizhou Provincial Environmental Science Research and Design Institute, Guiyang, China Guizhou Provincial Environmental Science Research and Design Institute Guiyang China; 5 Guizhou Caohai National Nature Reserve Management Committee, Bijie, China Guizhou Caohai National Nature Reserve Management Committee Bijie China

**Keywords:** Aquatic fungi, ascomycota, four new taxa, generic delimitation, molecular phylogeny

## Abstract

Three isolates of interest were collected during an investigation of freshwater fungi from wetlands in the karst regions of Guizhou Province, Southwest China. Phylogenetic analyses using ITS, LSU, SSU, and *tef-1α* gene regions have revealed the placements of these isolates in Melanommataceae (Pleosporales, Dothideomycetes). Based on the morphological and phylogenetic evidence, three new species are introduced: *Byssosphaeriachishuiensis***sp. nov.**, *Camposporiumguizhouense***sp. nov.**, and *C.aquaticum***sp. nov.***Byssosphaeriachishuiensis* is sister to *B.villosa* and forms a basal branch of *Byssosphaeria* within Melanommataceae. *Byssosphaeriachishuiense* is similar to *B.villosa* in ascal size but differs in the ascomata and ascospore sizes, and the ascospores lack appendages. *Camposporiumaquaticum* is sister to *C.guizhouense*, and they form a distinct lineage within the genus. Morphologically, *C.aquaticum* resembles *C.guizhouense* in its conidial shape but differs in conidial size. Additionally, PHI analysis is performed to further reveal that *C.aquaticum* and *C.guizhouense* have no significant recombination with related taxa. *Neobyssosphaeria* is synonymized under *Byssosphaeria*, and accordingly, *Byssosphaeriaclematidis***comb. nov.** is proposed to accommodate *Neobyssosphaeriaclematidis*. The descriptions, illustrations, and notes of the novel taxa are provided along with an updated phylogenetic tree of Melanommataceae. Two synopses of the species in *Byssosphaeria* and *Camposporium* are also provided.

## ﻿Introduction

The family Melanommataceae was introduced by Winter (1885) with *Melanomma* as the type genus. Melanommataceae members are characterized by globose or depressed perithecial ascomata, trabeculate pseudoparaphyses, bitunicate and fissitunicate asci, and pigmented and phragmosporous ascospores (Sivanesan 1984; Barr 1990; Zhang et al. 2012; Hyde et al. 2013; Tian et al. 2015; Li et al. 2017; Tennakoon et al. 2018). The trabeculate pseudoparaphyses is an important identification character of this family (Liew et al. 2000; Zhang et al. 2012; Hyde et al. 2013; Tian et al. 2015; Li et al. 2017; Tibpromma et al. 2017). Most species in this family have diverse lifestyles and habitats. They are fungicolous, hyperparasitic, parasitic, or saprobic and occur mainly on the twigs or bark of various woody plants in terrestrial, marine, and freshwater habitats in temperate and subtropical regions (Zhang et al. 2012; Hyde et al. 2013; Tian et al. 2015; Hashimoto et al. 2017; Wijayawardene et al. 2017; Beenken et al. 2020; Hongsanan et al. 2020). Mycologists have recently studied this family extensively (Mugambi and Huhndorf 2009; Hyde et al. 2013; Wijayawardene et al. 2014a, 2014b, 2017, 2020, 2022; Liu et al. 2015; Tian et al. 2015; Hyde et al. 2016; Tennakoon et al. 2018; Tibpromma et al. 2018; Beenken et al. 2020; Hongsanan et al. 2020; Bánki et al. 2023). Tian et al. (2015) reviewed Melanommataceae and accepted 20 genera in this family, including 13 sexual and 7 asexual genera. Subsequently, four genera, *Marjia*, *Melanocucurbitaria*, *Monoseptella*, and *Uzbekistanica*, were introduced by Wanasinghe et al. (2018a) based on the morphological and phylogenetic analyses. Koukol and Delgado (2021) reduced *Fusiconidium* and *Pseudobyssosphaeria* to synonymy with *Camposporium* and *Bertiella*, respectively. Recently, Gao et al. (2023) introduced *Dematiomelanomma*, resulting in 35 accepted genera in Melanommataceae, of which 28 genera have available sequence data (Koukol and Delgado 2021; Wijayawardene et al. 2022; Gao et al. 2023).

*Byssosphaeria* was introduced by Cooke and Plowright (1879) and typified by *B.keithii*. However, cultures and sequences of the type species are unavailable. *Byssosphaeria* spp. are characterized by superficial, separate, or gregarious ascomata, with a minute rounded ostiole and a coelomycetous asexual morph (Barr 1990; Hawksworth et al. 1995; Tennakoon et al. 2018). Asexual morphs have been reported in sporulated cultures as chaetophoma-like (Barr 1984) or pyrenochaeta-like coelomycetes (Samuels and Müller 1978). *Byssosphaeria* spp. are widespread, particularly on decorticated wood, bark of fallen branches, old leaves, and other plant substrates (Kirk et al. 2001; Zhang et al. 2012; Hyde et al. 2013; Tian et al. 2015; Tennakoon et al. 2018; Wanasinghe et al. 2018b; Kularathnage et al. 2022). Tennakoon et al. (2018) reviewed this genus and described two *Byssosphaeria* species (*B.macarangae* and *B.taiwanense*) in Melanommataceae based on multi-gene phylogenetic analyses. Index Fungorum (July 2024) listed 19 species. However, only 12 species have available sequence data (Tennakoon et al. 2018; Kularathnage et al. 2022).

*Camposporium* was introduced by Harkness (1884) and is typified by *C.antennatum*. The genus is polymorphic (Koukol and Delgado 2021) and characterized by dematiaceous, simple conidiophores and terminal, integrated, conidiogenous cells; the conidia are typically cylindrical and elongate, multi-septate, and rounded at one or both ends; the cells at each end are often paler in pigmentation than the central cells; the apex is either simple or has one or more cylindrical appendages; and the base typically has a persistent portion of the denticle attached (Hughes 1951; Ellis 1971; Ichinoe 1971; Whitton et al. 2002; Dong et al. 2020). Species of this cosmopolitan genus have been reported from freshwater and terrestrial habitats in many countries (Argentina, Australia, Brunei, Canada, China, Hawaii, Hungary, India, Japan, and Thailand) (Rao and Rao 1964; Dudka 1966; Ichinoe 1971; Matsushima 1971, 1983; Shearer 1974; Ellis 1976; Abdullah 1980; Castañeda 1985; Mercado et al. 1995; Whitton et al. 2002; Hyde et al. 2020a).

Most *Camposporium* spp. have cylindrical conidia with one or more appendages, and few have fusiform conidia, for example, *C.fusisporum* (Whitton et al. 2002), whereas others lack appendages (such as *C.ontariense* and *C.indicum*), which is reminiscent of those of *Fusiconidium* species. In a recent study, *Fusiconidium* was synonymized under *Camposporium* by Koukol and Delgado (2021) based on morphological comparisons (Koukol and Delgado 2021) and multigene phylogenetic analyses (Hyde et al. 2020a; Calabon et al. 2021). Twenty-five species epithets are listed in Index Fungorum (July 2024). However, sequence data are available for only 11 species.

During a survey of the taxonomy and diversity of freshwater fungi from karst plateau wetlands in Guizhou Province, China (Liu et al. 2019, 2020, 2023a, 2023b, 2023c; Yang et al. 2021, 2023), fresh collections were obtained from submerged decaying wood in freshwater and identified based on morphological and phylogenetic analyses. We introduce four novel species, *Byssosphaeriachishuiense* sp. nov., *B.clematidis* comb. nov., *Camposporiumaquaticum* sp. nov., and *C.guizhouense* sp. nov., with an illustrated account and molecular evidence. Their phylogenetic positions were determined based on maximum likelihood and Bayesian inference of combined ITS, LSU, SSU, and *tef-1α* sequence data.

## ﻿Materials and methods

### ﻿Specimen collection and examination

Specimens of submerged, decaying twigs were collected from wetlands in Guizhou Province, China, taken to the laboratory in zip-lock plastic bags, and incubated in plastic boxes lined with moistened, sterile filter paper at room temperature for one week. The Motic Nikon SMZ-171 (Nikon, Tokyo, Japan) dissecting microscope was used to observe fungal colonies and fruiting bodies. Fungal structures were examined and photographed using a Nikon ECLIPSE 80i compound microscope (Nikon) fitted with a Canon 70D digital camera (Canon, Tokyo, Japan). Single spores were isolated on water agar (**WA**), and germinated spores were transferred on potato dextrose agar (**PDA**) as described by Luo et al. (2018) and Senanayake et al. (2020). The Tarosoft Image Framework program was used for measurement, and the images used for photo plates were processed using Adobe Photoshop CS6 software. Herbarium specimens were deposited in the herbarium of the Guizhou Academy of Agriculture Sciences (**GZAAS**), Guiyang, China, and the herbarium of Cryptogams, Kunming Institute of Botany Academia Sinica (**HKAS**), Kunming, China. Axenic cultures were deposited to the Agricultural Culture Collection of China (**ACCC**) or Guizhou Culture Collection (**GZCC**). Facesoffungi and Index Fungorum numbers were registered as outlined by Jayasiri et al. (2015) and Index Fungorum (2024).

### ﻿DNA extraction, PCR amplification, and sequencing

Fungal mycelia were scraped using a sterilized scalpel and transferred to a 1.5 mL microcentrifuge tube for genomic DNA extraction with an Ezup Column Fungi Genomic DNA Purification Kit (Sangon Biotech, China). DNA was amplified by polymerase chain reaction (PCR). The ITS, LSU, SSU, and *tef-1α* gene regions were amplified using the primer pairs ITS5/ITS4 (White et al. 1990), LR0R/LR5 (Vilgalys and Hester 1990; Rehner and Samuels 1994), NS1/NS4 (White et al. 1990), and ef1-983F/ef1-2218R (Rehner and Buckley 2005), respectively, in a 25 μL reaction volume containing 9.5 μL ddH_2_O, 12.5 μL 2 × Taq PCR Master Mix with blue dye (Sangon Biotech, China), 1 μL of DNA template, and 1 μL of each primer (10 μM). The amplification condition consisted of initial denaturation at 94 °C for 3 min, followed by 40 cycles of 45 s at 94 °C, 50 s at 56 °C, and 1 min at 72 °C, and a final extension period at 72 °C for 10 min. The PCR products were purified and sequenced by Shanghai Sangon Biological Engineering Technology and Services Co. (Shanghai, China).

### ﻿Alignments and phylogenetic analyses

The ex-type and additional strains of Melanommataceae members were selected for phylogenetic analyses. The reverse and forward sequences generated in this study were assembled using DNAstar-SeqMan and subjected to BLASTn searches in the GenBank nucleotide database (http://blast.ncbi.nlm.nih.gov/, accessed on 12 May, 2024) to determine the most probable closely related taxa. The sequences were aligned using the online multiple alignment program MAFFT v.7 (http://mafft.cbrc.jp/alignment/server/, accessed on 12 May, 2024; Katoh and Standley 2013) and edited manually using BioEdit 7.2.3 (Hall 1999). The uninformative gaps and ambiguous regions were removed by trimAL v1.2 (http://trimal.cgenomics.org, accessed on 12 May, 2024; Capella-Gutiérrez et al. 2009) and manually combined in SequenceMatrix (Vaidya et al. 2011). The FASTA files were transferred to PHYLIP (for ML) and NEXUS (for BI) formats using the Alignment (ALTER) online program (Glez-Peña et al. 2010). Maximum likelihood (ML) analyses were performed on the CIPRES Science Gateway V. 3.3 (Miller et al. 2010). ML analyses were conducted with the RAxML-HPC v. 8.2.12 tool (Stamatakis 2014) using a GTRGAMMA approximation with rapid bootstrap analysis followed by 1000 bootstrap replicates. For the Bayesian inference (BI) approach, MrModeltest2 v. 2.3 (Nylander et al. 2008) was used to infer the appropriate substitution models. The GTR+G+I substitution model was selected for ITS, LSU, SSU, and *tef-1α* partitions. BI analyses were performed with MrBayes 3.2.6 (Ronquist et al. 2012). Six simultaneous Markov chains were run until the average standard deviation of the split frequencies was < 0.01, with trees saved every 1000 generations. The first 25% of saved trees, representing the burn-in phase, were discarded. The remaining trees were used to calculate the posterior probabilities (PP) of the recovered branches (Larget and Simon 1999).

The resulting trees were printed using FigTree v. 1.4.0 (Rambaut 2012), and the layout was edited using Adobe Illustrator 2019. The sequences generated in this study have been deposited in GenBank (Table 1).

**Table 1.** Taxa used in the phylogenetic analyses and their GenBank accession numbers. T denotes ex-type strains. Newly generated sequences and combined novel species are indicated in bold.

Taxon	Culture Accession No.	GenBank Culture Accession No.			
		ITS	LSU	SSU	*Tef-1α*
*Alpinariarhododendri*	CBS 141994^T^	KY189973	KY189973	KY190004	KY190009
*Aposphaeriacorallinolutea*	MFLU 15–2752	KY554202	KY554197	KY554200	KY554205
*Aposphaeriacorallinolutea*	MFLUCC 14–0504	–	KU243051	KU243052	KU243050
*Bertiellabambusae*	MFLUCC 18–0145^T^	MG737556	MG737555	–	MG737557
*Bertiellaellipsoidea*	MFLU 16–0583^T^	KX765261	KX765262	–	KX765263
*Bertiellafici*	NCYU 19–0073^T^	–	MW063224	MW079352	MW183787
*Bertiellamacrospora*	IL 5005^T^	–	GU385150	–	–
*Bertiellamacrospora*	SMH 3953^T^	–	–	–	GU327744
*Beverwykellapulmonaria*	CBS 283.53	KY189974	KY189974	KY190005	–
***Byssosphaeriachishuiense***	**ACCC 35242^T^**	**PQ164697**	**MW133847**	**MW134626**	**PQ231192**
***Byssosphaeriaclematidis***	**MFLUCC 17–0794^T^**	–	**MT214566**	**MT408594**	–
*Byssosphaeriadiffusa*	CBS 250.62^T^	–	DQ678071	DQ678019	DQ677915
*Byssosphaeriaguangdongense*	ZHKUCC 22–0335	OQ449320	OQ449288	OQ449337	–
*Byssosphaeriaguangdongense*	ZHKUCC 22–0336	OQ449321	OQ449289	OQ449338	–
*Byssosphaeriajamaicana*	SMH 1403^T^	–	GU385152	–	GU327746
*Byssosphaeriamacarangae*	MFLUCC 17–2655^T^	MH389782	MH389778	MH389780	MH389784
*Byssosphaeriamusae*	MFLUCC 11–0146^T^	KP744435	KP744477	KP753947	MH581149
*Byssosphaeriarhodomphala*	GKM L153N^T^	–	GU385157	–	GU327747
*Byssosphaeriasalebrosa*	SMH 2387^T^	–	GU385162	–	GU327748
*Byssosphaeriaschiedermayeriana*	GKM 152N^T^	–	GU385168	–	GU327749
*Byssosphaeriasiamensis*	MFLUCC 10–0099^T^	–	KT289895	KT289897	KT962059
*Byssosphaeriataiwanense*	MFLUCC17–2643^T^	MH389783	MH389779	MH389781	MH389785
*Byssosphaeriavillosa*	GKM 204N	–	GU385151	–	GU327751
*Camposporiumappendiculata*	DLUCC 1234^T^	MN758890	MN759021	MN758956	MN784094
***Camposporiumaquaticum***	**ACCC 35528^T^**	**PQ164699**	**MW133849**	**MW134628**	**PQ231194**
*Camposporiumatypicum*	NFCCI 4039	MF588672	MF588673	–	–
*Camposporiumcambrense*	FMR 12069	KY853428	KY853488	HF937343	–
***Camposporiumguizhouense***	**GZCC19–0480^T^**	**PQ164698**	**MW133848**	**MW134627**	**PQ231193**
*Camposporiumdulciaquae*	MFLUCC 21–0009^T^	MT864352	MT860430	MW485612	MW537104
*Camposporiumlycopodiellae*	CBS 143437^T^	MH107892	MH107939	–	–
*Camposporiummultiseptata*	DLUCC 792^T^	MN758889	MN759020	MN758955	MN784093
*Camposporiumpellucidum*	DLUCC 1239	MN758891	MN759022	MN758957	MN784095
*Camposporiumseptatum*	MFLUCC 19–0483^T^	NR168243	MN759023	NG068424	MN784096
*Camposporiumramosum*	CBS 132483	MH866030	MH877478	HF937344	–
*Camposporiumvaldivianum*	MFLUCC 14–0434^T^	–	KX611112	KX611114	KX611118
*Camposporiumverruculosum*	MFLUCC 16-0991^T^	–	KX641894	–	KX641896
*Cyclothyriellarubronotata*	CBS 121892^T^	KX650541	KX650541	–	KX650516
*Cyclothyriellarubronotata*	CBS 141486	KX650544	KX650544	KX650507	KX650519
*Dematiomelanommayunnanense*	KUNCC 23–12728^T^	OQ225528	OQ360647	OQ360651	OQ413238
*Dematiomelanommayunnanense*	KUNCC 23–12730	OQ225529	OQ360648	OQ360652	OQ413239
*Herpotrichiajuniperi*	CBS 200.31^T^	–	DQ678080	DQ678029	DQ677925
*Herpotrichiamacrotricha*	SMH 269	–	GU385177	–	GU327756
*Herpotrichiaxiaokongense*	KUMCC 21–0004^T^	–	MZ408889	MZ408891	MZ394066
*Herpotrichiavaginatispora*	MFLUCC 13–0865^T^	–	KT934252	KT934256	KT934260
*Marjiatianschanica*	TASM 6120^T^	MG828909	MG829019	MG829126	MG829206
*Marjiauzbekistanica*	TASM 6122^T^	MG828911	MG829021	MG829128	MG829208
*Melanocamarosporioidesugamica*	MFLUCC 17–2314^T^	MH000192	MH000190	MH000191	MH006610
*Melanocucurbitariauzbekistanica*	MFLUCC 17–0829^T^	MG828912	MG829022	MG829129	MG829209
*Melanodiplodiatianschanica*	TASM 6111^T^	MG828914	MG829023	MG829130	MG829210
*Melanodiplodiatianschanica*	MFLUCC 17–0805^T^	MG828913	MG829025	MG829132	MG829212
*Melanommajaponicum*	CBS 142905^T^	–	LC203338	LC203292	LC203367
*Melanommapopulicola*	CBS 543.70	NR170056	NG075164	NG070237	–
*Melanommapulvispyrius*	CBS 124080^T^	–	GU456323	GU456302	GU456265
*Monoseptellarosae*	MFLUCC 17–0815^T^	MG828916	MG829026	MG829133	MG829213
*Monoseptellarosae*	TASM 6114	MG828917	MG829027	MG829134	MG829214
*Muriformistrickeriarosae*	MFLU 16–0227^T^	MG828918	MG829028	MG829135	MG829215
*Muriformistrickeriarubi*	MFLUCC 17–2550	MG828919	MG829029	MG829136	MG829216
*Muriformistrickeriarubi*	MFLUCC 15–0681^T^	–	KT934253	KT934257	KT934261
*Petrakiaechinata*	WU 36922	KY189980	KY189980	KY190007	KY190015
*Petrakiaechinata*	CBS 133070	JQ691628	LC203352	LC203306	LC203380
*Phragmocephalaatra*	MFLUCC 15–0021^T^	KP698721	KP698725	KP698729	–
*Phragmocephalagarethjonesii*	MFLUCC 15–0018^T^	KP698722	KP698726	KP698730	–
*Phragmotrichumchailletii*	CPC 33263^T^	MN313812	MN317293	–	–
*Pleotrichocladiumopacum*	AU BD04	JN995638	JN941370	JN938733	–
*Pleotrichocladiumopacum*	FMR 12416^T^	KY853462	KY853523	–	–
*Praetumpfiaobducens*	WU 36895	KY189982	KY189982	–	KY190017
*Praetumpfiaobducens*	CBS 141474^T^	KY189984	KY189984	KY190008	KY190019
*Pseudostrickeriamuriformis*	MFLUCC13–0764^T^	–	KT934254	KT934258	KT934262
*Pseudostrickeriaononidis*	MFLUCC14–0949^T^	–	KT934255	KT934259	KT934263
*Pseudostrickeriarosae*	MFLUCC 17–0643^T^	MG828954	MG829065	MG829169	MG829234
*Pseudotrichiamutabilis*	WU 36923	KY189988	KY189988	–	KY190022
*Pseudotrichiamutabilis*	SMH 1541	–	GU385209	–	–
*Sarimanaspseudofluviatile*	MAFF 239465^T^	LC001717	LC001714	LC001711	–
*Sarimanasshirakamiense*	HHUF 30454^T^	NR138017	NG059803	NG061263	–
*Seifertiaazalea*	ZTMyc 59953^T^	MK502003	MK502026	MK502037	MK502083
*Seifertiashangrilaensis*	MFLUCC 16–0238^T^	–	KU954100	KU954101	KU954102
*Tumulariaaquatica*	CBS 212.46^T^	MH856165	MH867689	–	–
*Tumulariatuberculata*	CBS 256.84	–	GU301851	–	GU349006
*Uzbekistanicapruni*	MFLU 17–2136^T^	MN758893	MN759024		MN784097
*Uzbekistanicarosaehissaricae*	MFLUCC 17–0819^T^	MG828975	MG829087	MG829187	MG829242
*Uzbekistanicayakutkhanika*	MFLUCC 17–0842^T^	MG828978	MG829090	MG829190	MG829245
*Xenostigminazilleri*	CBS 115685	GU269840	GU253857	–	–

## ﻿Results

### ﻿Phylogenetic analyses

**Figure 1.**
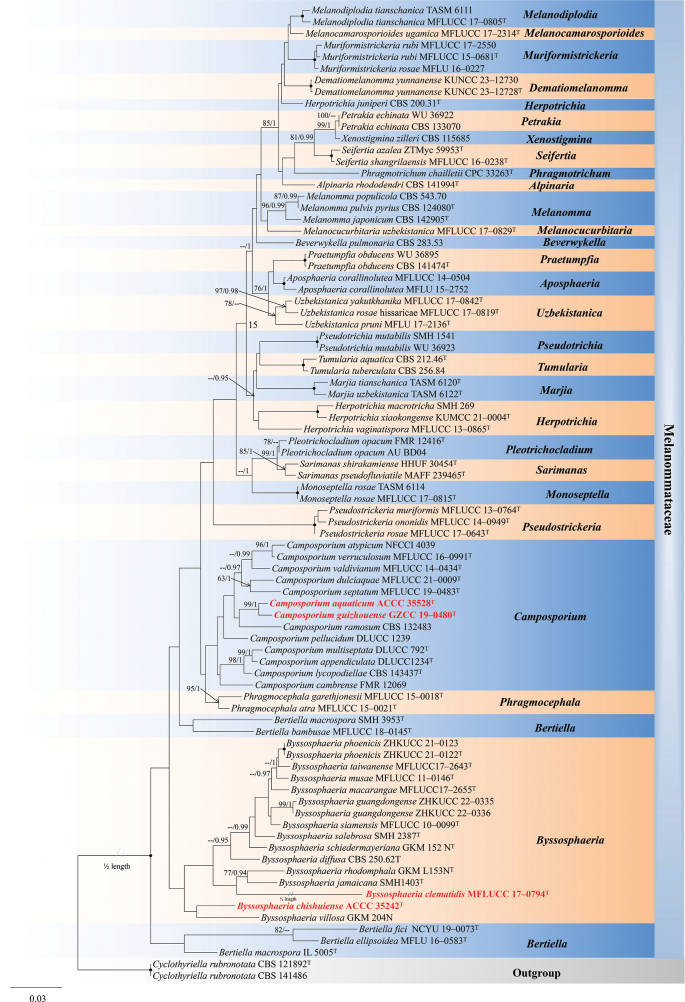
Maximum likelihood majority rule consensus tree for Melanommataceae using ITS, LSU, SSU, and *tef1-α* sequence data. Bootstrap support values for maximum likelihood (ML) greater than 75% and Bayesian posterior probabilities greater than 0.95 are indicated above branches as ML-BS/PP. The scale bar represents the expected number of changes per site. The tree is rooted with *Cyclothyriellarubronotata* (CBS 121892^T^ and CBS 141486). Ex-type strains are indicated with a superscript^T^. The new taxa are in red and bold. Branches with 100% ML-BS and 1.0 PP were dotted with black dots. Genera are indicated as colored blocks.

The phylogenetic analyses of Melanommataceae isolates using combined ITS, LSU, SSU, and *tef-1α* sequences showed the three new strains represented three distinct lineages in the *Byssosphaeria* and *Camposporium* subclades (Fig. 1). The alignment comprised 84 strains with 3194 nucleotides (1–437, 438–1274, 1275–2274, and 2275–3194 bp of ITS, LSU, SSU, and *tef-1α*, respectively), including gaps. *Cyclothyriellarubronotata* (CBS 121892 and CBS 141486) served as the outgroup taxa. The best-scoring RAxML tree is shown in Fig. 1. The analyzed ML and Bayesian trees were topologically similar and did not conflict significantly.

The phylogenetic analyses of Melanommataceae showed that *Byssosphaeria* species clustered together to form a monophyletic subclade without significant support. The new isolate ACCC 35242 was sister to *B.villosa* (GKM 204N) with weak support. The two new isolates, GZCC19-0480 and ACCC 35528, clustered together within *Camposporium* with strong statistical support (99% ML BS/1.00 PP; Fig. 1) and were sister to *Camposporiumramosum* (CBS 132483) with weak support.

### ﻿Taxonomy

#### ﻿ Byssosphaeria chishuiense

Taxon classificationFungiPleosporalesMelanommataceae

L.L. Liu & Z.Y. Liu sp. nov.

B97415BF-E375-5EAF-9CD9-C215E5F33DAC

Index Fungorum: IF902545

Facesoffungi Number: FoF15497

Fig. 2

##### Etymology.

Refers to the Chishui River basin, where the holotype was collected.

##### Holotype.

GZAAS 20-0374.

##### Description.

***Saprobic*** on decaying, submerged wood in freshwater habitats. ***Sexual morph*. *Ascomata*** superficial, gregarious, unilocular, globose to subglobose, 220–390 μm high, 520–740 μm diam., broadly or narrowly conical, black, carbonaceous, roughened and irregular, ostiolate. ***Ostiole*** single, central, papillate. ***Peridium*** 33–58 μm wide at sides, brown, thick-walled, of ***textura angularis***, comprising a mixture of host and fungal cells. ***Hamathecium*** 2.5–3.5 μm wide, composed of dense, trabeculate, distinctly septate, anastomosing, pseudoparaphyses, embedded in a hyaline gelatinous matrix. ***Asci*** 111–163(–220) × 14–24.5 μm (x̄ = 145 × 19 μm, n = 20), 8-spored, bitunicate, fissitunicate, cylindrical clavate, short pedicel, apically round, with an ocular chamber. ***Ascospores*** overlapping, 1–2-seriate, fusiform, hyaline, 1-septate, constricted at the central septum, lower cell wider and longer than upper cell, 41.5–51.5 × 7–9 μm (x̄ = 45.5 × 8 μm, n = 30), tapering to pointed apices, mostly straight, surrounded by a thick distinctive sheath, 4–6.3 μm wide (in water-mounted slide), drawn out at the ends, smooth-walled. ***Asexual morph*.** Undetermined.

##### Cultural characteristics.

Conidia germinated on WA, and germ tubes were produced from both ends within 12 h. Colonies on PDA overgrew the culture dish after 3 weeks at 25 °C in dark, circular, mycelia dense in the middle and sparse in the edge, white from above, pale yellow from reverse.

##### Material examined.

China • Guizhou Province, Chishui River basin, near 28°25'N, 106°0'E, at 500 m altitude, saprobic on submerged decaying wood in a freshwater stream, July 2019, L.L. Liu, CS1-13 (GZAAS 20-0374, holotype); • ex-type living culture ACCC 35242.

##### Taxonomic notes.

**Figure 2.**
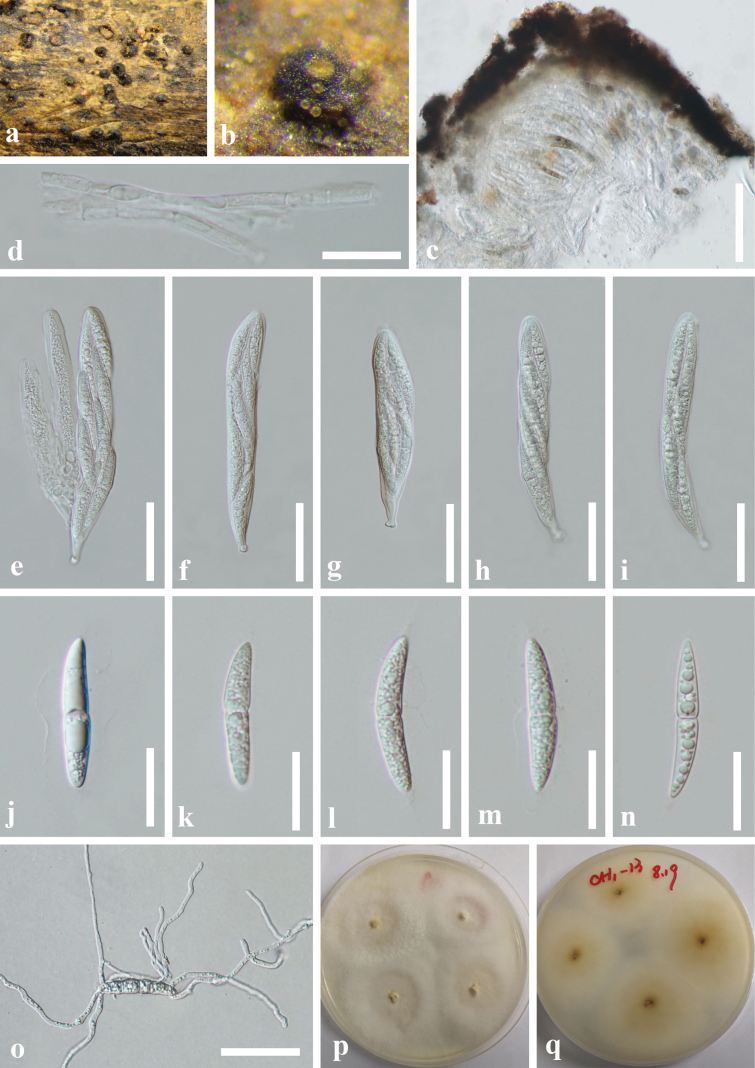
*Byssosphaeriachishuiense* (GZAAS 20-0374, holotype) **a, b** ascomata on natural substrate **c** section of an ascoma **d** pseudoparaphyses **e–i** asci **j–n** ascospores **o** germinated ascospore **p, q** culture from above and below, respectively. Scale bars: 50 μm (**b–o**).

Morphologically, *B.chishuiense* is characterized by superficial ascomata with bright yellow or orange flat apices around the ostiole and hyaline ascospores (Fig. 2). These characteristics are consistent with the generic concept of *Byssosphaeria* (Barr 1990). Phylogenetic analyses of the combined LSU, SSU, ITS, and *tef-1α* sequence data showed that *B.chishuiense* (ACCC 35242) was sister to *B.villosa* (GKM 204N), and they reside within the Melanommataceae (Fig. 1). Although *B.chishuiense* and *B.villosa* have the same-sized asci (140–200 × 17–25 vs. 140–200 × 17–25 μm), *B.chishuiense* can be distinguished from *B.villosa* in having larger ascospores (41.5–51.5 × 7–9 vs. 30–40 × 8–12 μm) and ascomata (520–740 vs. 430–500 μm diam.) and the absence of appendages (Samuels and Müller 1978). The main morphological differences between *Byssosphaeria* spp. are detailed in Table 2. Additionally, comparing 828 nucleotides across the LSU gene region between *B.chishuiense* and *B.villosa* revealed 29 bp (3.50%) differences. Our molecular data also support that *B.chishuiense* and *B.villosa* are phylogenetically distinct (Fig. 1).

**Table 2.** Synopsis of *Byssosphaeria* species.

Species	Ascomata Size high × diam. (μm)	Asci Size (μm)	Ascospores			References
			Size (μm)	Color	Septa	
*Byssosphaeriaalnea*	220–460 diam.	105–140 × 7.5–12	19–24 × 4–5	Pale brown	1–3	Barr (1984)
***B.chishuiense***	**220–390 × 520–740**	**111–163 (–220) × 14–24.5**	**41.5–51.5 × 7–9**	**Hyaline**	**1–2**	**This study**
*B.diffusa*	340–480 × 300–450	80–110 × 9–12	16–20 × 4–6	Pale brown	1	Chen and Hsieh (2004)
*B.erumpens*	420–520 × 450–550	105–130 × 12–15	20–25 × 5–6	Dark brown	1	Chen and Hsieh (2004)
*B.erythrinae*	–	–	22–28 × 6–7.5	Hyaline to palebrown	1–3	Barr (1984)
*B.hainanensis*	182–291 × 165–280	115–130 × 7–11	10.5–14 × 3.5–5	Pale brown	1	Li and Zhuang (2008)
*B.jamaicana*	340–550 diam.	80–120 × 12–15	25–35 × 7–8	Light to clearbrown	1–3	Barr (1984)
*B.juniper*	220–400 diam.	100–145 × 12–18	18–27 × 6–9	Pale brown	3–4	Barr (1984)
*B.keithii*	350–520(−600) × (320−)400–620	80–112(−150)×10–13	80–112(−150) × 10–13	pale brown	1	Berkeley and Broome 1876; Tian et al. (2015)
*B.macarangae*	450–550 × 575–650	75–100 × 7–10	20–25 × 4–5	Hyaline	1(−3)	Tennakoon et al. (2018)
*B.musae*	430–540 × 450–630	125–135 × 12–14	30–33 × 5–6	Hyaline to light brown	1–3	Liu et al. (2015)
*B.oviformis*	1500–2000 × 1000–1500	120–130 × 7–9	25–30 × 2.5–3.5	Hyaline	1	Barr (1984)
*B.phoenicis*	580–625 × 600–650	100–160 × 10–15	25–30 × 5–7	Brick red to brown	3	Kularathnage et al. (2022)
*B.rhodomphala*	220–500 diam.	85–120 × 10–13	18–23 × 6–7.5	Light brown	1–(3)	Barr (1990)
*B.salebrosa*	440–800 diam.	120–150 × 13–16.5	40–50 × 7–9	Hyaline to light brown	1–3(−5)	Barr (1984)
*B.schiedermayeriana*	500–825; 423–511 × 478–537	100–150 × 12–15	32–42 × 5–8	Light brown	1–3(−5)	Barr (1984)
*B.semen*	400–600 × 330–550	80–110 × 9–12	20–30 × 3.5–4.5	Hyaline to pale brown	1–3	Barr (1984); Tian et al. (2015)
*B.siamensis*	501–692 × 561–720	112–148 × 10–16	40.5–50 × 7–11	Hyaline to pale yellow	1(−3)	Tian et al. (2015)
*B.taiwanense*	460–540 × 450–500	125–150 × 12–14	30–35 × 7–8	Pale to olivaceous brown	1	Tennakoon et al. (2018)
*B.villos*a	340–470 × 430–500	140–200 × 17–25	30–40 × 8–12	Hyaline to brown	1–2	Boise (1984); Samuels and Müller (1978)
*B.xestothele*	330–440 × 330–550	70–100 × 9–12	20–26 × 4.5–6	Hyaline to pale brown	1–3	Barr (1984)

#### ﻿ Byssosphaeria

Taxon classificationFungiPleosporalesMelanommataceae

Cooke, Grevillea 7 (43): 84 (1879)

692B6DCB-962F-5C1B-B03B-41E3B842055F

##### Synonym.

*Neobyssosphaeria* Wanas., E.B.G. Jones & K.D. Hyde, Fungal Diversity 102: 57 (2020).

#### ﻿ Byssosphaeria clematidis

Taxon classificationFungiPleosporalesMelanommataceae

(Wanas., Phukhams., E.B.G. Jones & K.D. Hyde) L.L. Liu & Z.Y. Liu comb. nov.

7D600585-F8C6-52AB-91B9-2CAADCC30C34

Index Fungorum: IF557190

Facesoffungi Number: FoF07282

##### Basionym.

*Neobyssosphaeriaclematidis* Wanas, Phukhams., E. B. G. Jones, & K. D. Hyde, Fungal Diversity 102:57 (2020).

##### Material examined.

UK • Hampshire, Botleywood, on dead stems of *Clematisvitalba*, 25 May, 2016, E.B.G. Jones, GJ 298 (MFLU 17-0614, holotype); • ex-type living culture, MFLUCC 17-0794.

##### Description.

Phukhamsakda et al. (2020).

##### GenBank accession numbers.

LSU: MT214566; SSU: MT408594.

##### Taxonomic notes.

Phukhamsakda et al. (2020) introduced *Neobyssosphaeria*, which was grouped with *Byssosphaeria* to form a basal lineage in their phylogenetic analyses of combined LSU, SSU, and ITS sequence data. Morphologically, Phukhamsakda et al. (2020) distinguished *Neobyssosphaeria* from *Byssosphaeria* by immersed ascomata with central papilla filled with periphyses, cellular pseudoparaphyses, and broad fusiform and hyaline ascospores. However, these characters also exist in some *Byssosphaeria* species. For example, *B.siamensis* has ascomata with cellular pseudoparaphyses (Tian et al. 2015), and other species, such as *B.salebrosa* and *B.villosa*, have broad, fusiform, and hyaline ascospores (Samuels and Müller 1978; Barr 1984), being reminiscent of *Neobyssosphaeriaclematidis* (Phukhamsakda et al. 2020). The only distinctive features are immersed ascomata in *Neobyssosphaeria* and superficial ascomata in *Byssosphaeria*. We regard it as not sufficient for the separation of these two genera (Sandoval-Denis et al. 2016; Sun et al. 2023). Additionally, in our phylogenetic analyses, *Neobyssosphaeriaclematidis* formed an internal clade within *Byssosphaeria*. Therefore, we transfer *Neobyssosphaeriaclematidis* to *Byssosphaeria* as *B.clematidis* and treat *Neobyssosphaeria* as a synonym of *Byssosphaeria*.

#### ﻿ Camposporium aquaticum

Taxon classificationFungiPleosporalesMelanommataceae

L.L. Liu & Z.Y. Liu sp. nov.

B2DCD996-3D7D-5E2C-B02D-382C8DDA919D

Index Fungorum: IF902546

Facesoffungi Number: FoF16381

Fig. 3

##### Etymology.

Refers to the aquatic habitat of this fungus.

##### Holotype.

GZAAS 20-0376.

##### Description.

***Saprobic*** on decaying, submerged wood in a freshwater habitat. ***Asexual morph*. *Colonies*** on natural substrate effuse, brown, procumbent. ***Mycelium*** partly immersed and partly superficial, composed of pale brown, septate, cylindrical hyphae. ***Conidiophores*** macronematous, mononematous, irregularly cylindrical, strongly flexuous to twisted, procumbent, brown, sometimes fading slightly towards the apex, smooth-walled, unbranched, thick-walled, 4–8–septate, 75–92 × 5.5–6.5 μm (x̄= 86 × 6 μm, n = 20). ***Conidiogenous cell***: holoblastic, monoblastic, integrated, terminal, cylindrical, pale brown. ***Conidia*** solitary, cylindrical to narrowly fusoid, elongate, subhyaline to pale olivaceous green when young, brown or pale brown when mature, polar cells slightly paler, 64–114 × 7.5–11.5 μm (x̄= 91.5 × 9.5 μm, n = 30), smooth-walled, verruculose, 6–13-septate, often slightly constricted at the septa, basal cell conical, apical cell rounded with 2 independent, simple, cellular, aseptate, hyaline, smooth, straight, curved, or flexuous appendages, 29.5–40 × 1.5–2.5 μm (x̄= 33 × 1.8 μm, n = 20). ***Sexual morph*.** Undetermined.

##### Cultural characteristics.

Conidia germinated on WA within 24 h, and germ tubes were produced from the apex. The colonies on PDA reached approximately 22 mm diam. after 3 weeks at 25 °C under dark conditions. Colonies circular, with raised center and filamentous edge, grey at center, brown at the edge from above, dark brown in the center, brown at the edge from reverse.

**Figure 3.**
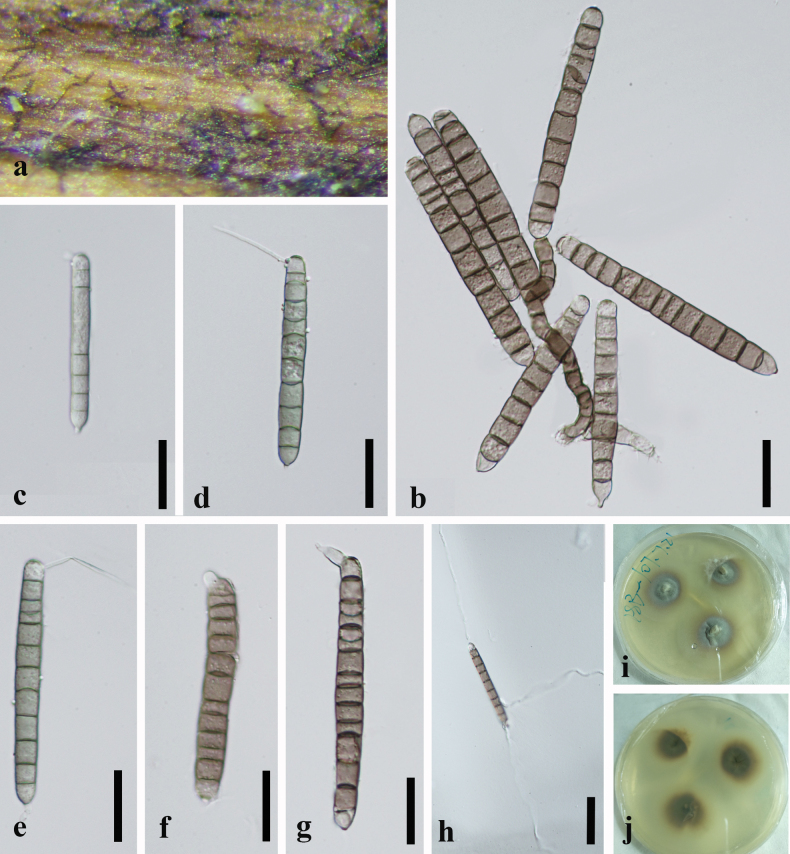
*Camposporiumaquaticum* (GZAAS 20-0376, Herbarium) **a** colonies on natural substrate **b** conidiophore and conidium **c–g** conidia **h** germinating conidia **i, j** culture **i** from above **j** from below. Scale bars: 25 μm (**c–j)**; 100 μm (**h)**.

##### Material examined.

China • Guizhou Province, Guiyang City, Baihua Lake, near 26°39'18.20"N, 106°32'12.90"E, at 1205 m altitude, on a decaying branch submerged in the lake, 18 April, 2018, Lingling Liu, 18B-107 (GZAAS 20-0376, holotype); • ex-type living culture, ACCC 35528.

##### Taxonomic notes.

**Figure 4.**
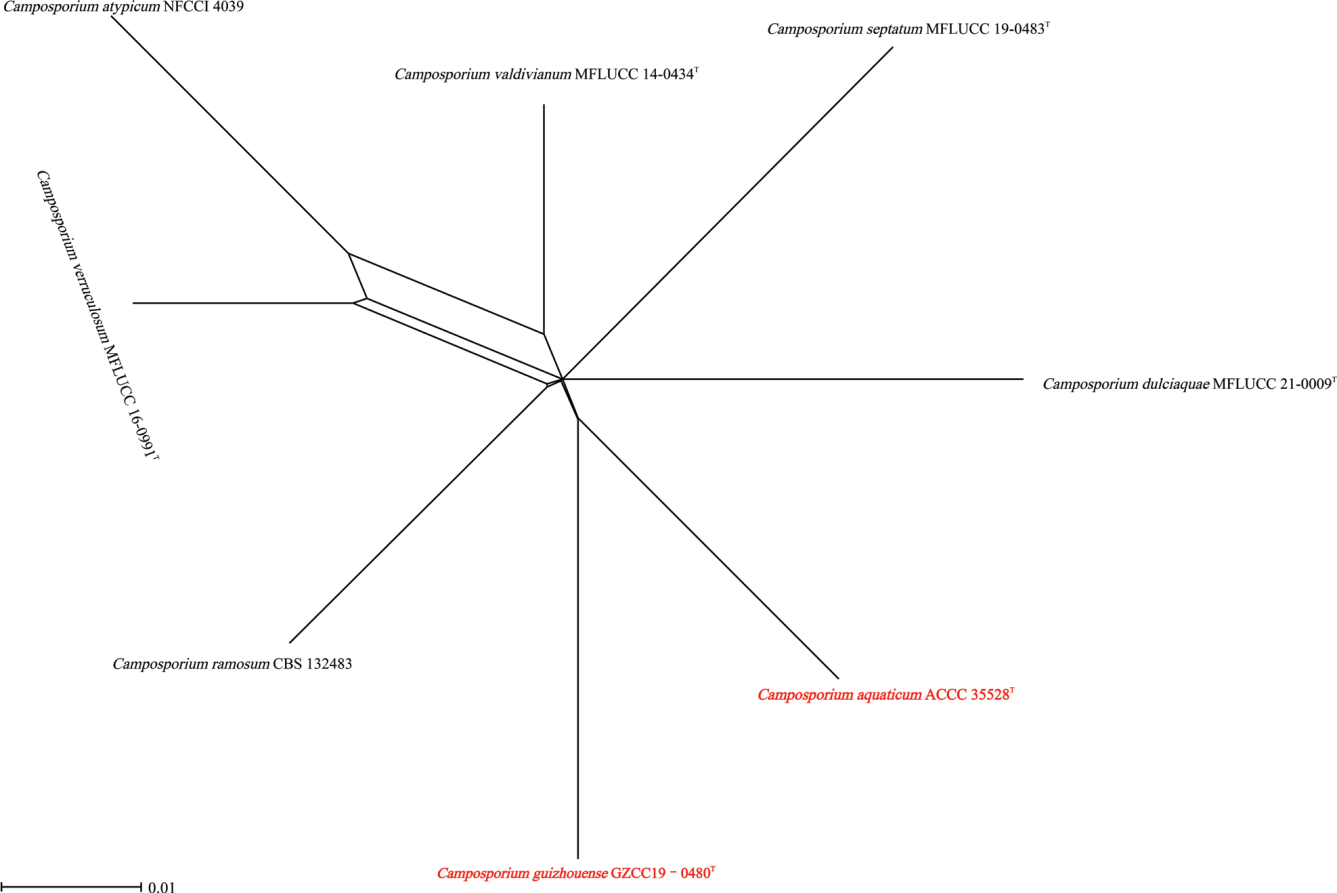
Split graphs showing the results of the PHI test of *C.aquaticum* (ACCC 35528^T^) with their most closely related species (Fw = 0.9994). The new taxa are shown in red bold.

Phylogenetic analyses of the combined LSU, SSU, ITS, and *tef1-α* sequence dataset showed that *C.aquaticum* (ACCC 35528) is sister to *C.guizhouense* (GZCC 19-0480) and formed a distinct lineage. We compared the base pair differences between the two new taxa and found six base pair differences (1.27%) across 471 nucleotides in the ITS gene region. Additionally, comparing the 972 nucleotides across the *tef1-α* gene region between ACCC 35528 and GZCC 19-0480 shows a 17 base pair difference (1.75%). Morphologically (Table 3), *C.aquaticum* (ACCC 35528) conidia resemble *C.guizhouense* conidia. However, *C.aquaticum* conidia (64–114 × 7.5–11.5 μm) are substantially larger than *C.guizhouense* conidia (58–81.5 × 7–9 μm), and *C.aquaticum* conidia are 6–13-septate, whereas *C.guizhouense* conidia are 8–11-septate (mostly 10). PHI analysis further confirmed that *C.aquaticum* (ACCC 35528) showed no significant genetic recombination with closely related species (Fw > 0.05, Fig. 4). Therefore, we introduce *C.aquaticum* (ACCC 35528) as a novel species.

**Table 3.** Synopsis of *Camposporium* species.

Species name	Conidiophores		Conidia			Appendage			References
	Size (μm)	Septation	Size (μm)	Septation	Colour	Morphology	Septation	Length (μm)	
*Camposporiumantennatum*	76–166 × 6	Up to 12	42–78 × 7.5–8.8	7–14 (4–14)	Pale–brown, paler end cells	1–3(simple)	Aseptate	Up to 40	Harkness (1884)
*C.appendiculatum*	very short	Septate	107–119 × 9.5–11.5	10–13	Pale–brown, hyaline at both end cells	1(simple)	Aseptate	72–114	Hyde et al. (2020)
***C.aquaticum***	**75–92 × 5.5–6.5 μm**	**4–8**	**64–114 × 7.5–11.5 μm**	**6–13**	**Brown, paler end cells**	**1 (simple)**	**Septate**		**This study**
*C.atypicum*	150–685 × 5–10	Septate	35–42 × 13–15	5–7	Brown, paler end cells	0	–	–	Koukol and Delgado (2021)
*C.cambrense*	22–84 × 6–7	3–10	62–115 × 8–10	Up to 15	Pale–brown, paler end cells	1(simple)	Septate	32–126	Hughes (1951)
*C.dulciaquae*	16–95 × 5–9	Septate	100–130 × 8.5–13	8–11	Brown, paler end cells	(2–)3 (simple)	Aseptate		Calabon et al. (2021)
*C.fusisporum*	100–145 × 6.5–10	10–15	86–115 × 13.5–19	8–11	Brown, paler end cells	2–3 (simple)	Aseptate	17–40	Whitton et al. (2002)
***guizhouense***	**60–110 × 4–6.5**	**3–9**	**58–81.5 × 7–9**	**8–11**	**Pale–brown, paler end cells**	**2 (simple)**	**Aseptate**	**24.5–47.5**	**This stud**y
*C.hyalinum*	10–40 × 4–6	0–1	20–75 × 3–5	2–6	Hyaline, concolourous	1 (simple)	Aseptate	16–55	Abdullah (1980)
*C.hyderabadense*	25.2–39.6 × 3.6–5.4	1–3	32.4–54 × 3.6–7.2	5–9	Dark–brown, concolourous	1–4 (simple)	Aseptate	Up to 43.2	Rama and Rao (1964)
*C.indicum*	28.5–50.4 × 3.6–7.2	2–5	21.6–72 × 3.6–7.2	3–14	Dark–brown, paler end cells	0	–	–	Rama and Rao (1964)
*C.japonicum*	37.5–77.5 × 5–6.5	5	42.5–70 × 5–7.5	7–10	Pale–brown, concolourous	0–1 (2–4–branched)	Aseptate	Up to 36	Ichinoe (1971)
*C.laundonii*	Up to 40 × 5–8	0–2	50–150 × 13–17	4–9	Brown, paler end cells	1–2 (simple)	Septate	Up to 60	Ellis (1976)
*C.lycopodiellae*	20–50 × 3–5	1–2	(65–)75–85(–100) × (7–)8(–9)	(7–)8–9(–11)	Pale–brown, hyaline at both end cells	0	–	–	Crous et al. (2018); Hyde et al. (2020a)
*C.marylandicum*	41–127 × 2–3	0–5	24.7–44 × 4.5–6.5	5–10	Hyaline, concolourous	1 (simple)	?	33.5–80	Shearer (1974)
*C.microsporum*	Up to 72 × 3.6–7.2	1–5	25.8–36 × 7.2–9	2–6	Pale–brown to brown, concolourous	1–2 (simple)	Aseptate	10.8–28.8	Rama and Rao (1964)
*C.multiseptata*	short	Septate	97–111 × 9–11	10–13	Dark–brown, paler end cells	1 (simple)	Septate	11–17	Hyde et al. (2020a)
*C.ontariense*	45–200 × 5–7	6–8	20–35 (20–53) × 8–12 (6.5–12)	3–7 (3–9)	Pale–brown, concolourous	0	–	–	Matsushima (1983)
*C.pellucidum*	30 to 150 × 5–8	Up to 10	78–140 × 7.5–12	Up to 16	Pale–brown, paler end cells	1(simple)	Septate	30–145	Hughes 1951; Hyde et al. (2020a)
*C.quercicola*	15–60 × 3.5–4	1–3	28–45 × 3.5–4.5	5–9	Pale–brown, paler end cells	0–3 (simple)	Aseptate	Up to 30	Mercado et al. (1995)
*C.ramosum*	70–138 × 5.2–6	4–10	80–112 × 6.4–9.6	8–15	Brown, paler end cells	1(1–3–branched)	1–2 septate	20–60	Whitton et al. (2002)
*C.septatum*	–	Septate	98–125 × 7–11.5	(8–)9 (–11)	Median brown, paler end cells	(2–)3 (simple)	Aseptate	–	Hyde et al. (2020a)
*scolecosporium*	10–20 × 2.5–4.5	0–3	48–108 × 3–4	6–12	Hyaline, concolourous	0	–	–	Kobayasi (1971)
*C.valdivianum*	91–114 × 8.5–11	7–9	38–40 × 12–15	7	Pale brown to dark brown, paler end cells	0	–	–	Li et al. (2017); Koukol and Delgado (2021)
*C.verruculosum*	49–65 × 5.5–7.5	1–3	59–66 × 15–18	8–10	Drown to dark brown, paler end cells	0	–	–	Li et al. (2017); Koukol and Delgado (2021)

#### ﻿ Camposporium guizhouense

Taxon classificationFungiPleosporalesMelanommataceae

L.L. Liu & Z.Y. Liu sp. nov.

73479268-9575-5089-BF9B-E2D1EAF605A2

Index Fungorum: IF902547

Facesoffungi Number: FoF15498

Fig. 5

##### Etymology.

Refers to the location where the holotype was collected.

##### Holotype.

GZAAS 20-0375.

##### Description.

***Saprobic*** on decaying, submerged wood in freshwater habitats. ***Asexual morph*. *Colonies*** on natural substrates are velvety, effuse, hairy, scattered, brown, and glistening. ***Mycelium*** partly immersed, partly superficial, hyaline to pale brown, septate, and cylindrical. ***Conidiophores*** macronematous, mononematous, irregularly cylindrical, straight to flexuous or twisted, erect, pale to mid-brown, sometimes fading slightly towards the apex, smooth-walled, unbranched, 3–9-septate, 60–110 × 4–6.5 μm (x̄ = 80 × 5 μm, n = 20). ***Conidiogenous cells*** were holoblastic or polyblastic, integrated into the apical region of the conidiophore, denticulate, cylindrical, pale brown, smooth-walled, and sometimes attached to the conidia following detachment. ***Conidia*** solitary, cylindrical to narrowly fusoid, elongate, brown or pale brown, 58–81.5 × 7–9 μm (x̄ = 70 × 8 μm, n = 20), paler at both ends, verrucose, thickened walls, 8–11-septate (mostly 10), basal cell conical with a truncate end, apical cell rounded with two independent, simple, cellular, aseptate, hyaline, smooth, straight, curved, or flexuous appendages, 24.5–47.5 × 1.5–3.5 μm (x̄ = 35 × 2 μm, n = 20). ***Sexual morph*.** Undetermined.

##### Cultural characteristics.

Conidia germinated on WA within 24 h, and germ tubes were produced from the apex. Colonies on PDA reached approximately 25 mm diam. after 3 weeks at 25 °C in dark, circular, grey-white, or yellowish mycelium, dense in the middle and sparse in the edge, in reverse, pale brown to brown, smooth in the margin.

**Figure 5.**
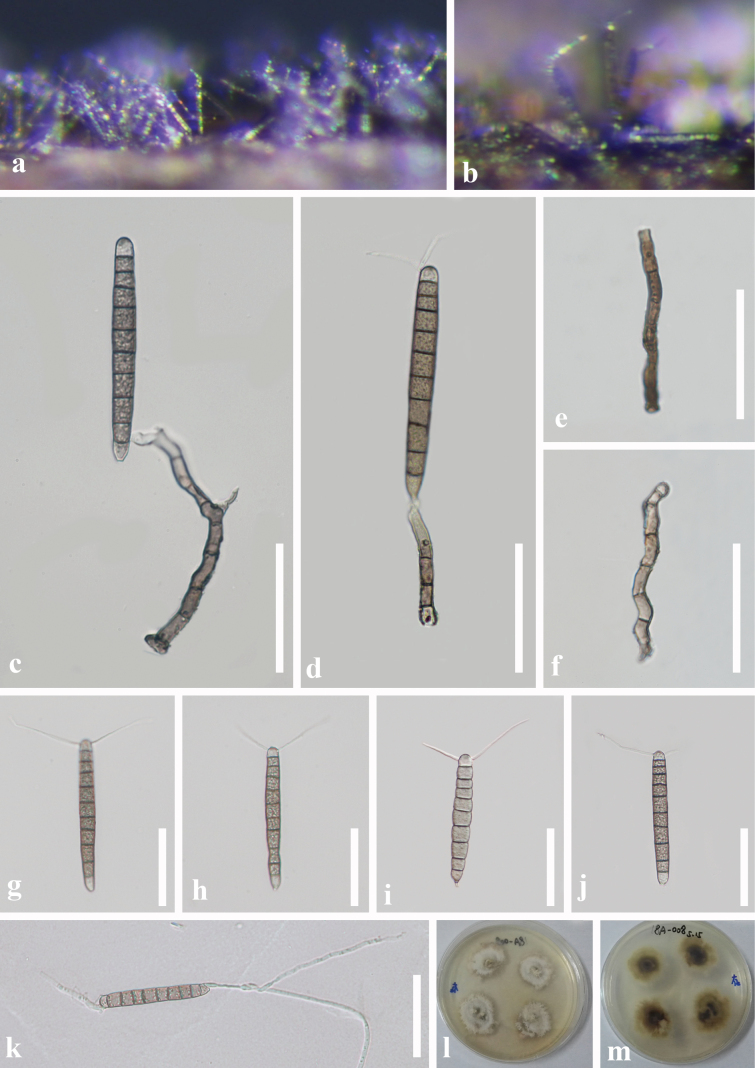
*Camposporiumguizhouense* (GZAAS 20-0375, Herbarium) **a, b** colonies on natural substrate **c, d** conidiophore and conidium **e, f** conidiophores **g–j** conidia **k** germinated conidium **l, m** culture **l** from above **m** from below. Scale bars: 50 μm (**c–j**).

##### Material examined.

China • Guizhou Province, Guiyang City, Aha Lake, near 26°32'N, 106°40'E, at 1085 m altitude, on a decaying branch submerged in the lake, 16 April, 2018, L.L. Liu, 18A-8 (GZAAS 20-0375, holotype); • ex-type living culture, GZCC 19-0480.

##### Taxonomic notes.

Phylogenetic analyses of the combined LSU, SSU, ITS, and *tef1-α* sequence dataset showed that *C.guizhouense* was sister to *C.ramosum* (CBS 132483) and formed a distinct lineage. We compared the *ITS* sequences of the new taxon (GZCC 19-0480) to that of *C.ramosum* (CBS 132483). A difference of 7.87% (37/470 bp) was observed. Additionally, comparing 812 nucleotides across the LSU gene region between GZCC 19-0480 and *C.ramosum* (CBS 132483) revealed 13 base pair differences (1.60%). Morphologically (Table 3), *C.guizhouense* conidia resemble *C.dulciaquae*, *C.fusisporum*, *C.ramosum*, and *C.septatum* conidia in shape. However, the conidia of *C.guizhouense* (58–81.5 × 7–9 μm) are considerably smaller than those of *C.dulciaquae*, *C.fusisporum*, *C.ramosum*, and *C.septatum* (100–130 × 8.5–13, 98–125 × 7–11.5, 80–112 × 6.4–9.6, and 86–115 × 13.5–19 μm, respectively). Moreover, the conidia of *C.guizhouense* have two simple and aseptate apical appendages; those of *C.dulciaquae*, *C.fusisporum*, and *C.septatum* have 2–3 apical appendages, and those of *C.ramosum* have 1–3 simple or branched septate appendages. Therefore, we introduce our collection as a new species, *C.guizhouense*.

## ﻿Discussion

Over the last 20 years, freshwater ascomycetes have been extensively researched (Ranghoo and Hyde 1998; Ranghoo et al. 2000; Zhang et al. 2013; Luo et al. 2019; Calabon et al. 2020; Dong et al. 2020; Yang et al. 2023). Interestingly, molecular data were only used to resolve species in the early 2000s (Tsui et al. 2001; Jeewon et al. 2003; Kodsueb et al. 2004; Shearer et al. 2009, 2014; Hu et al. 2012; Hyde et al.2013; Boonmee et al. 2014; Cai et al. 2014; Yang et al. 2018). With the recent increasing availability and accessibility of molecular data, the number of identified species has increased rapidly, particularly in China and Thailand (Luo et al. 2018; Dong et al. 2020; Yang et al. 2023). Zhang et al. (2011) listed 173 species belonging to 112 genera recorded from freshwater habitats. Hu et al. (2013) reported 782 freshwater fungal species in China. Luo et al. (2019) identified 451 species belonging to 160 genera of freshwater Sordariomycetes. Dong et al. (2020) provided 145 genera of freshwater Dothideomycetes (six orders, 43 families), of which 32% (46 genera) were unique to freshwater habitats (Calabon et al. 2020; Dong et al. 2020), and Liu (2020) introduced 102 freshwater fungal species from 68 genera in karst wetlands in Guizhou Province, China. Recently, Yang et al. (2023) reported 47 new taxa, 7 new combinations, 10 new geographical records, and 9 habitat records collected from karst regions in China and Thailand. To the best of our knowledge, 68 new taxa of freshwater fungi have been reported in the karst regions of Guizhou Province, China (Liu et al. 2020; Yang et al. 2023). In this study, three novel taxa of freshwater ascomycetes from karst wetlands in Guizhou Province, China, were introduced based on combined multilocus phylogeny and morphological approaches. It appears that numerous new taxa await being discovered and described, as new freshwater habitats are explored, or a particular genus is being studied with molecular data, wherein its diversity is much higher than previously anticipated in China (Luo et al. 2019; Bao et al. 2020; Dong et al. 2020; Hyde et al. 2020b; Yang et al. 2023).

In this study, multi-gene phylogenetic analyses of Melanommataceae, including 28 genera with available sequence data for molecular comparisons, showed that *Byssosphaeria* and *Camposporium* are closely related and nested within Melanommataceae. *Byssosphaeriaschiedermayeriana* is the first freshwater species in *Byssosphaeria* reported from Seychelles (Barr 1984), and this species has also been reported to exist in terrestrial habitats (Tian et al. 2015). Our species is the second freshwater species in *Byssosphaeria*. Contrarily, *Camposporium* species are more frequently found in freshwater habitats. The new taxon, *C.guizhouense* is the fifth freshwater species in this genus, and other 14 species recorded in freshwater habitats are *C.antennatum* (Rajashekhar and Kaveriappa 1996; Tsui et al. 2001; Smits et al. 2007), *C.appendiculatum* (Hyde et al. 2020a), *C.cambrense* (Gonczol and Revay 2004; Kurniawati et al. 2010; Gonczol and Revay 2011), *C.dulciaquae* (Calabon et al. 2021), *C.fusisporum* (Cai et al. 2003), *C.hyalinum* (Abdullah 1980), *C.japonicum* (Gonczol and Revay 2004; Gonczol and Revay 2011), *C.marylandicum* (Shearer 1974; Ingold 1976; Gonczol and Revay 1998), *C.multiseptatum* (Hyde et al. 2020a), *C.ontariense* (Gonczol and Revay 2004), *C.pellucidum* (Ingold 1976; Gonczol and Revay 1998; Gonczol and Revay 2004; Czeczuga et al. 2005; Smits et al. 2007; Gonczol and Revay 2011; Hyde et al. 2020a), *C.quercicola* (Cai et al. 2003), *C.septatum* (Hyde et al. 2020a), and *C.verruculosum* (Li et al. 2017; Koukol and Delgado 2021).

Phylogenetic analyses of Melanommataceae showed that *B.chishuiense* and *B.villosa* formed an independent and distinct lineage sister to other basal *Byssosphaeria* species. *Byssosphaeriavillosa* was transferred from *Herpotrichia* (Samuels and Müller 1978), and its placement was confirmed subsequently (Mugambi and Huhndorf 2009; Tennakoon et al. 2018). The phylogenetic placement of *Byssosphaeria* in this study is similar to previous publications (Liu et al. 2015; Tian et al. 2015; Tennakoon et al. 2018), but its monophyly is not well supported, especially when *B.chishuiense* and *B.villosa* are involved. We temporarily accept these two species in *Byssosphaeria*, but more fresh collections are needed to resolve the taxonomic confusions of *Byssosphaeria*.

However, multi-gene phylogenetic analyses of Melanommataceae, which included all *Camposporium* strains, showed that *Camposporium* and *Fusiconidium* were grouped together within Melanommataceae. The placement of these two genera is consistent with the results of previous studies (Calabon et al. 2020; Hyde et al. 2020a). However, Calabon et al. (2020) and Hyde et al. (2020a) treated them as distinct genera based on morphological differences in conidiogenic cells and conidial shapes. Subsequently, Koukol and Delgado (2021) advocated that *Fusiconidium* is synonymous with *Camposporium* and listed numerous morphological similarities between them. For instance, *C.fusisporum* has fusiform conidia (Whitton et al. 2002), while the conidia of *C.ontariense* and *C.indicum* lack appendages. Moreover, *F.indicum* (Pratibha et al. 2017) has polyblastic conidiogenous cells with sympodial proliferation and slender, denticle-like separating cells, suggesting that these characteristics are similar to those of some species of *Camposporium* (Pratibha et al. 2017; Koukol and Delgado 2021). In this study, we accept that *Fusiconidium* combined *Camposporium*, *Fusiconidiumaquaticum*, and *F.indicum* were renamed *C.verruculosum* and *C.atypicum*; *Fusiconidiumlycopodiellae* and *F.mackenziei* were combined with *C.lycopodiellae* and *C.valdivianum* (Hyde et al. 2020a; Koukol and Delgado 2021).

## Supplementary Material

XML Treatment for Byssosphaeria chishuiense

XML Treatment for Byssosphaeria

XML Treatment for Byssosphaeria clematidis

XML Treatment for Camposporium aquaticum

XML Treatment for Camposporium guizhouense

## ﻿Additional information

### Author ORCIDs

Ling-Ling Liu https://orcid.org/0000-0003-0006-0938

Ya-Ya Chen https://orcid.org/0000-0002-8293-168X

Jiu-Lan Gou https://orcid.org/0000-0001-6439-158X

Xiao-Feng Gu https://orcid.org/0000-0002-1197-2070

Quan-Quan Wei https://orcid.org/0000-0002-1672-1588

Meng Zhang https://orcid.org/0000-0002-3312-1402

Zuo-Yi Liu https://orcid.org/0000-0001-5348-8458

### Data availability

The datasets generated for this study are available in the NCBI database.
